# Effects of genomic copy number variants penetrant for schizophrenia on cortical thickness and surface area in healthy individuals: analysis of the UK Biobank

**DOI:** 10.1192/bjp.2020.139

**Published:** 2021-02

**Authors:** Xavier Caseras, George Kirov, Kimberley M. Kendall, Elliott Rees, Sophie E. Legge, Matthew Bracher-Smith, Valentina Escott-Price, Kevin Murphy

**Affiliations:** 1 MRC Centre for Neuropsychiatric Genetics and Genomics, Division of Psychological Medicine and Clinical Neurosciences, Cardiff University, UK; 2 MRC Centre for Neuropsychiatric Genetics and Genomics, Division of Psychological Medicine and Clinical Neurosciences, Cardiff University; and UK Dementia Research Institute, Cardiff University, UK; 3 Cardiff University Brain Research Imaging Centre (CUBRIC), School of Physics and Astronomy, Cardiff University, UK

**Keywords:** Imaging, genetics, schizophrenia, copy number variation, cortical anatomy

## Abstract

**Background:**

Schizophrenia is a highly heritable disorder with undetermined neurobiological causes. Understanding the impact on brain anatomy of carrying genetic risk for the disorder will contribute to uncovering its neurobiological underpinnings.

**Aims:**

To examine the effect of rare copy number variants (CNVs) associated with schizophrenia on brain cortical anatomy in a sample of unaffected participants from the UK Biobank.

**Method:**

We used regression analyses to compare cortical thickness and surface area (total and across gyri) between 120 unaffected carriers of rare CNVs associated with schizophrenia and 16 670 participants without any pathogenic CNV. A measure of cortical thickness and surface area covariance across gyri was also compared between groups.

**Results:**

Carrier status was associated with reduced surface area (β = −0.020 mm^2^, *P* < 0.001) and less robustly with increased cortical thickness (β = 0.015 mm, *P* = 0.035), and with increased covariance in thickness (carriers *z* = 0.31 *v.* non-carriers *z* = 0.22, *P* < 0.0005). Associations were mainly present in frontal and parietal areas and driven by a limited number of rare risk alleles included in our analyses (mainly 15q11.2 deletion for surface area and 16p13.11 duplication for thickness covariance).

**Conclusions:**

Results for surface area conformed with previous clinical findings, supporting surface area reductions as an indicator of genetic liability for schizophrenia. Results for cortical thickness, though, argued against its validity as a potential risk marker. Increased structural thickness covariance across gyri also appears related to risk for schizophrenia. The heterogeneity found across the effects of rare risk alleles suggests potential different neurobiological gateways into schizophrenia's phenotype.

Schizophrenia is a highly heritable (*h*^2^ ~ 80%),^1^ severe and chronic psychiatric disorder for which we have greatly advanced our understanding of the genetic risk factors;^2,3^ however, this has not been followed by a significant improvement in our understanding of its neurobiological underpinnings. Our knowledge about how genetic risk alleles for schizophrenia affect brain anatomy is still scarce. Recent research looking into rare risk alleles, such as copy number variants (CNVs), has provided some clues. However, most of these studies have suffered from low statistical power and/or have included participants with severe psychopathology, adding the confounding effect of variables related to the presence of mental illness (i.e. medication or increased alcohol intake) known to have an impact on brain anatomy.^4–6^ Since genetically driven brain changes would be present – albeit attenuated – in healthy at-risk participants, we aimed to investigate the effects of schizophrenia- associated (SCZ-associated) CNVs on cortical thickness and surface area in a sample of unaffected participants, therefore avoiding the above confounders. Cortical thickness and surface area have been shown to be under different genetic influences in adults and to capture different neurobiological aspects of brain development, which justifies evaluating them separately rather than in a combined measure of volume.^7^ We were also interested in looking at the effect of SCZ-associated CNVs on cortical thickness and surface area structural covariances. Structural covariance networks are heritable^8^ and reflect patterns of maturational trajectories,^9^ therefore providing potential valuable information on the neurobiological substrates of schizophrenia.

Considering previous research in clinical samples,^10^ and assuming that genetically driven brain changes will also express, albeit in an attenuated form, in unaffected SCZ-associated CNV carriers, we hypothesised that this group will present with thinner cortex and reduced surface area compared with unaffected non-carriers, these differences being larger in frontal and temporal regions. We also hypothesised that SCZ-associated CNV carriers will present with altered structural covariance compared with non-carriers, although owing to the scarcity and inconsistency of results regarding cortical thickness and surface area covariation,^11,12^ we did not make any *a priori* assumption about the direction of this effect. We also aimed to examine whether any effects of SCZ-associated CNVs over cortical anatomy were generalisable across individual CNVs or whether individual CNVs had distinctive effects over cortical anatomy.

## Method

### Participants

This study used a subsample of participants from the UK Biobank (www.ukbiobank.ac.uk) for which brain magnetic resonance imaging (MRI) T1 images were available at the time of running the analyses (*n* = 20 664). All participants have consented to take part in genetic and imaging studies. The UK Biobank was granted ethical approval by the North West Multi-Centre Research Ethics Committee. Data were released to us under project reference 17044.

Only participants reporting White British or Irish descent and for whom genetic analysis confirmed European ancestry^13^ (*n* = 18 534) were included. Furthermore, we excluded participants with personal history of severe neuropsychiatric disorders or medical/neurological conditions that could affect cortical anatomy, using self-reported diagnosis made by a doctor at any assessment visit or from hospital records, as follows: self-reported from category 20002, including codes for other substance abuse/dependency (1410), opioid dependency (1409), alcohol dependency (1408), mania/bipolar disorder/manic depression (1291), schizophrenia (1289), dementia/alzheimers/cognitive impairment (1263), Parkinson's disease (1262), multiple sclerosis (1261), motor neuron disease (1259), chronic/degenerative neurological problem (1258); and hospital records from categories 41202 and 41204, including ICD-10 codes for Down syndrome (Q90), pervasive developmental disorders (F84), mental retardation categories (F71, F72, F73, F78, F79, F81), manic disorder/bipolar affective disorder (F30, F31), other psychosis (F21, F22, F23, F28, F29), schizoaffective disorders (F25), disorders due to psychoactive substance use (F11–F19), disorders due alcohol use (F10), dementia (F00, F01, F02, F03, F04, G30, R54), multiple sclerosis (G35–G37), neurodegenerative disorders (G11, G13, G23, G31, G32), Alzheimer's disease (G30), Parkinson's disease (G20–G22), motor neuron disease (G122) and Huntington's disease (G10). After applying these criteria, the sample size was reduced to 18 214 participants.

### Genotyping and CNV calling

Genotyping was performed using the Affymetrix UK BiLEVE Axiom® array (807 411 probes) on an initial 50 000 participants, and the Affymetrix UK Biobank Axiom® array (820 967 probes) for the remaining participants. The two arrays are very similar (with over 95% common content). Sample processing at UK Biobank is described in their documentation (https://biobank.ctsu.ox.ac.uk/crystal/docs/genotyping_sample_workflow.pdf).

CNV calling was conducted following the same procedure as described in a previous study.^14^ Briefly, normalised signal intensity, genotype calls and confidences were generated using ~750 000 biallelic markers that were further processed with PennCNV-Affy software running in UNIX.^15^ Individual samples were excluded if they had >30 CNVs, a waviness factor >0.03 or <−0.03 or call rate <96%. After this quality control, the final sample was reduced to *n* = 17 234. Individual CNVs were excluded if they were covered by <10 probes or had a density coverage of <1 probe per 20 000 base pairs.^14^

A list of the CNVs that so far have been significantly associated with schizophrenia^3,16–18^ and the genomic coordinates of their critical regions can be found in supplementary Table 1, available at https://doi.org/10.1192/bjp.2020.139, along with their breakpoints. These were manually inspected to confirm that they met our CNV calling criteria. Briefly, we required a CNV to cover more than half of the critical interval and to include the key genes in the region (if known) or, in the case of single-gene CNVs, the deletions to intersect at least one exon and the duplications to cover the whole gene. As a control comparison, we used individuals who carried none of the 93 CNVs that have previously been associated with neurodevelopmental disorders^19,20^ (non-carriers). The criteria for defining pathogenic CNVs have been previously fully described.^14^ Applying these criteria excluded from our analyses 444 participants who carried at least one of these pathogenic CNVs that have not yet been robustly associated with increased risk for schizophrenia, bringing the total sample to *n* = 16 790 (120 carrying SCZ-associated CNVs and 16 670 non-carriers).

### Brain imaging data

Brain images were acquired using Siemens Skyra 3 T scanners in UK Biobank's imaging centres in Cheadle (*n* = 14 154) and Newcastle (*n* = 2636) using identical acquisition protocols.^21^ T1-weighted brain images were processed using FreeSurfer v.5.3 software running in UNIX (https://surfer.nmr.mgh.harvard.edu) to automatically obtain estimates of mean cortical thickness (mm) and surface area (mm^2^) for the whole brain and for each gyrus, based on the Desikan–Killiany (D–K) atlas^22^ parcellation included in FreeSurfer. To avoid error values due to deficient segmentation of tissue types or parcellation into gyri, extreme values (defined as ±2.5 standard deviations from the group mean) were removed from the analyses. Mean surface area and cortical thickness were slightly but significantly different between recruitment centres (*t*(16 564) = 5.15, *P* < 0.001 and *t*(16 494) = 6.43, *P* < 0.001 respectively); therefore, ‘centre’ was added as a covariate in subsequent analyses.

### Analyses

Regression analyses including age, gender, intracranial volume and centre as covariates were run to examine the association between cortical measures and CNV carrier status. We first compared carriers of any SCZ-associated CNV with non-carriers on overall brain and gyri mean surface area and cortical thickness, to subsequently examine the effect of each individual SCZ-associated CNV present in our sample (only 6 SCZ-associated CNVs were present in >2 carriers and were therefore analysed independently; Table 1).

**Table 1:** Association between specific copy number variant (CNV) carrier status and cortical surface area and thickness^a^

CNV and number of carriers	Surface area, mm^2^		Thickness, mm	
	Total *n*	β (*P*)	Total *n*	β (*P*)
1q21.1 del (*n* = 9)	16 456	**−0.011 (0.006)**	16 389	0.009 (0.198)
1q21.1 dup (*n* = 11)	16 459	−0.001 (0.858)	16 391	0.001 (0.933)
*NRXN1* del (*n* = 6)	16 454	−0.007 (0.101)	16 386	−0.001 (0.850)
15q11.2 del (*n* = 59)	16 506	**−0.016 (<0.001)**	16 437	**0.020 (0.006)**
16p13.11 dup (*n* = 25)	16 473	−0.007 (0.088)	16 404	−0.003 (0.626)
16p12.1 del (*n* = 8)	16 456	−0.006 (0.148)	16 388	0.008 (0.261)

del, deletion; dup, duplication.

a. β-values were assigned to CNV carrier status after regressing out the effect of gender, age, intracranial volume and scanning centre. *P*-values that survived false discovery rate (FDR) correction based on the 12 tests included in this table are highlighted in bold.

To investigate structural covariance, *z*-transformed correlation matrices were generated for thickness and surface area separately. For each pair of gyri in the D–K atlas, the *z*-transformed correlation across carriers was calculated. For a given gyrus, the average of the *z*-transformed correlations with all other gyri provided a measure of ‘integration’ for that gyrus. An overall brain structural covariance measure was calculated by averaging that metric across all gyri. Additionally, two independent component analyses (ICAs) were run for surface area and cortical thickness, setting the number of components at 20. The mean structural covariance within each of those components was calculated by weighting each gyrus's covariance by its relative weight in the ICA mixing matrix and taking the average across all gyri. To compare these covariance metrics between groups, null distributions were generated using a random sampling procedure. A group of non-carriers the same size as the carrier group was randomly selected and structural covariance was calculated as above separately for surface area and cortical thickness. This was repeated 100 000 times, generating a null distribution for each pairwise gyrus comparison. An empirical *P*-value could then be derived by counting the number of null samples in which the absolute structural covariance value was greater than the absolute value in the carriers.

Significance threshold was set at *P* < 0.05 (two-sided), and a false discovery rate (FDR) of 0.10 (based on Benjamini–Hochberg^23^) was used to correct for multiple testing. For the overall brain's surface area and cortical thickness, FDR correction was applied on the basis of the 14 tests performed. For within-gyri analyses, FDR correction was applied separately to each CNV group comparison; i.e. FDR was based on the 136 tests that resulted from comparing surface area and cortical thickness within each gyrus across both hemispheres between carriers (or each individual CNV) and non-carriers.

## Results

Gender was evenly split (53% female) and equally distributed across carriers and non-carriers (χ^2^(1) = 0.78, *P* > 0.1). The mean age was 55 years (s.d. = 7.46, range = 45–80), not differing between groups (*t*(16 788) = 0.07, *P* > 0.1). Among carriers, 49% carried the most common 15q11.2 deletion CNV; five of the rarer targeted CNVs were not present in our sample (see supplementary Table 1). Previous work from our group has shown consistency of individual CNV frequencies between UK Biobank batches, and between the UK Biobank and other independent control data-sets.^14^ As shown in previous research,^14,24^ SCZ-associated CNV carrying status predicted performance IQ (fluid intelligence) (β = −0.04, *P* = 4 × 10^−7^) and scores on the Townsend deprivation index (β = 0.02, *P* = 0.019) in both cases, with carriers being disadvantaged.

### Total brain surface area and cortical thickness

Carrying any SCZ-associated CNV was associated with surface area reductions (β = −0.020 mm^2^, *P* < 0.001), but thicker cortex (β = 0.015 mm, *P* = 0.035). However, analyses on single CNVs showed only the 1q21.1 deletion and the 15q11.2 deletion to be associated with reduced surface area in carriers; and only the latter was also associated with thicker cortex in carriers (Table 1).

### Gyrus surface area and cortical thickness

**Fig. 1:**
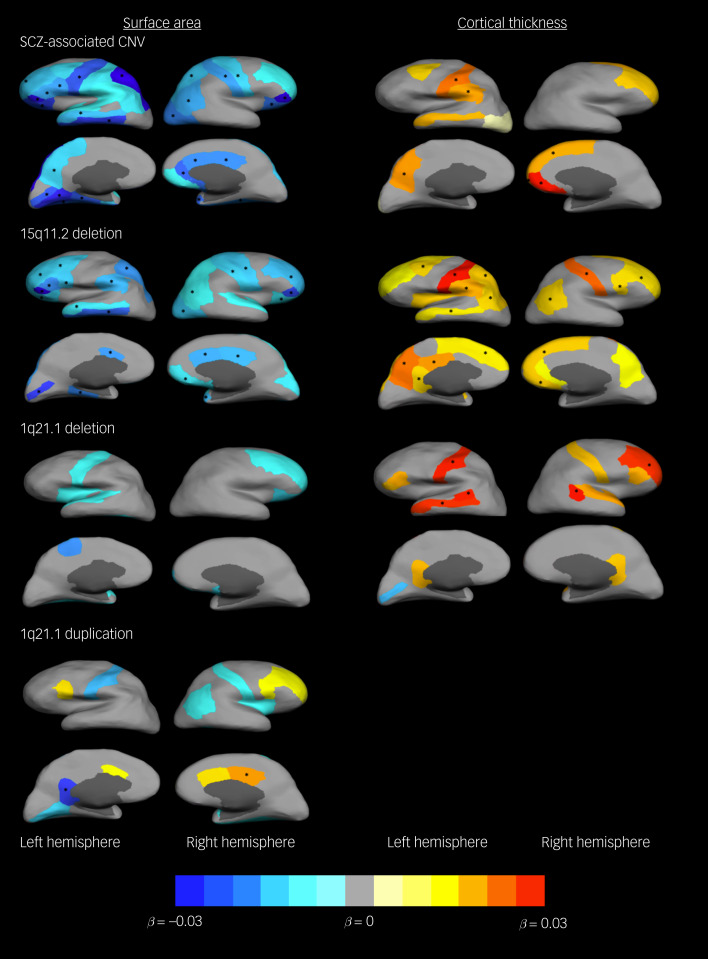
Differences in cortical surface area (mm^2^) and thickness (mm) between carriers of schizophrenia-associated copy number variants (SCZ-associated CNVs) and those not carrying CNVs, and carriers of individual SCZ-associated CNVs and those not carrying CNVs. Gyri with nominal significant associations are coloured: warm colours for positive associations (carriers larger) and cold colours for negative associations (carriers smaller). Gyri in which the association with CNV carrier status remained significant after false discovery rate (FDR) correction are marked with an asterisk (*). Only SCZ-associated CNVs that presented with FDR-corrected significant associations are included in the figure (1q21.1duplication did not present any significant association with cortical thickness even before FDR correction).

Schizophrenia-associated CNV carrier status was associated with surface area across several gyri bilaterally, although again associations appeared mainly driven by the 15q11.2 deletion. In all cases, carriers showed reduced surface area (Fig. 1 and supplementary Table 2a). Only one other SCZ-associated CNV, i.e. 1q21.1 duplication, showed FDR-corrected association with surface area. In this case, though, it indicated reduced surface area in left isthmus cingulate in carriers, but increased surface area in right posterior cingulate. It is worth noting that left and right anterior cingulate along with dorsal prefrontal areas also showed nominal larger surface area in carriers of the 1q21.1 duplication (Fig. 1 and supplementary Table 2(a)). In fact, this was the only SCZ-associated CNV that showed positive associations with surface area, with all other SCZ-associated CNVs showing only negative associations at either FDR-corrected or nominal levels of significance (supplementary Table 2(a)).

The results for cortical thickness showed fewer significant associations, mostly in right frontal cortex and left parietal regions. Again, these results appeared mainly driven by the 15q11.2 deletion, which showed more widespread significant effects when analysed independently. The 1q21.1 deletion was also associated with thickness in left postcentral gyrus, middle temporal gyrus and banks of the superior temporal sulcus, and rostral middle frontal gyrus. In all cases, carriers presented thicker cortices (Fig. 1 and supplementary Table 2(b)). No other FDR-corrected associations were found (supplementary Tables 2(a) and 2(b)).

To examine the effect of overall brain size in the above results, we repeated these analyses excluding intracranial volume as a covariate. This only slightly changed the results for cortical thickness but had a significant impact on results for surface area. The 15q11.2 deletion lost its association with mean surface area (supplementary Table 3), as well as seeing a reduction in effect size for most of the previously significant associations with gyri's surface area (supplementary Table 4). The 1q21.1 deletion and duplication showed stronger associations – negative and positive respectively – with mean surface area and surface area across different gyri, this effect being more noticeable in the deletion CNV that in this reanalysis showed significant associations with most brain gyri (supplementary Tables 3 and 4).

To exclude the possibility that our results are confounded by population stratification, we repeated our main analyses including the first ten principal components derived from common alleles as covariates, obtaining the same results as before.

### Structural covariance

**Fig. 2:**
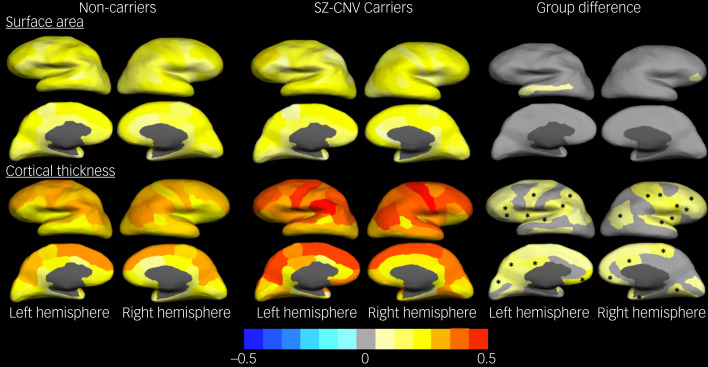
Structural covariance index (averaged *z*-transformed correlation with all other gyri) for cortical surface area and thickness for carriers of schizophrenia-associated copy number variants (CNVs) and non-carriers, and difference between groups. In all cases the colour scheme at the bottom indicates the effect size (z-value for averaged maps, difference in z-value under ‘group difference’). Gyri with nominal significant associations are coloured: warm colours for positive associations (carriers larger) and cold colours for negative associations (carriers smaller). Gyri in which the association with CNV carrier status remained significant after false discovery rate (FDR) correction are marked with an asterisk (*).

Overall structural covariance for surface area was not different between groups (carriers *z* = 0.16 versus non-carriers *z* = 0.14, *P* = 0.239), this being the case across all gyri (Fig. 2 and supplementary Table 5(a)) and across the 20 ICA components (supplementary Fig. 1). Structural covariance for cortical thickness, though, was globally increased in carriers compared with non-carriers (*z* = 0.31 *v. z* = 0.22, *P* < 0.0005), this difference appearing widely spread across gyri (Fig. 2 and supplementary Table 5(b)) and across 14 of the 20 ICA components (supplementary Fig. 1).

**Fig. 3:**
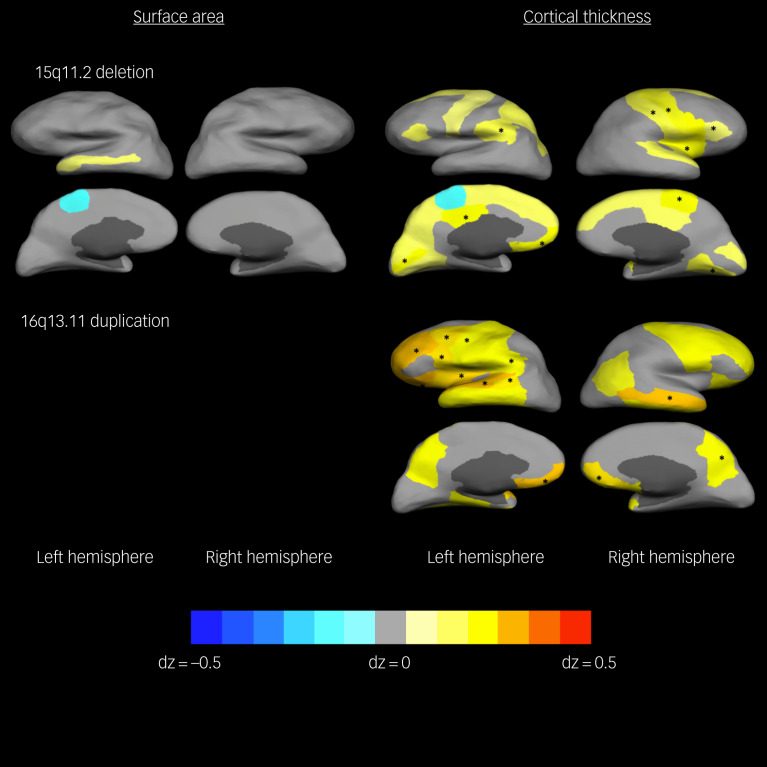
Differences in cortical surface area and thickness covariance between carriers of individual schizophrenia-associated copy number variants (SCZ-associated CNVs) and those not carrying CNVs. Gyri with nominal significant associations are coloured: warm colours for positive associations (carriers larger) and cold colours for negative associations (carriers smaller). Gyri where the association with CNV carrier status remained significant after false discovery rate (FDR) correction are marked with an asterisk (*). Only SCZ-associated CNVs that presented with FDR-corrected significant associations are included in the figure (16p13.11duplication did not present any significant association for surface area covariances even before FDR correction).

As regards individual SCZ-associated CNVs, the 15q11.2 deletion and the 16p13.11 duplication were associated with increased cortical thickness covariance mainly in frontal and parietal cortices (Fig. 3 and supplementary Table 5(b)). No other CNV showed FDR-corrected association with cortical thickness covariance, and none of them did with surface area covariance. These results were replicated comparing carrier groups on the structural covariance across ICA components. Again the 15q11.2 deletion and 16p13.11 duplication showed increased thickness covariance across half the components compared with non-carriers, and those mainly included gyri within temporal and parietal lobules. The 1q21.1 duplication also showed a pattern of increased thickness covariance relative to non-carriers similar to that for the aforementioned two CNVs. No component showed group differences as regards surface area covariance (supplementary Fig. 1).

## Discussion

We report here significant associations between SCZ-associated CNV carrier status and different indices of cortical anatomy. Surface area appeared reduced in SCZ-associated CNV carriers, following our prediction based on previous research in large clinical samples – supporting this as a potential marker of risk for schizophrenia. Cortical thickness, though, showed the opposite effect than predicted; this is, thicker cortex in carriers. Structural covariance for cortical thickness appeared to be increased in carriers, indicating lack of flexibility or modularity across gyri. However, owing to the novelty of our structural covariance approach this result would require further replication. Finally, our results suggest large heterogeneity of effects across individual SCZ-associated CNVs, suggesting that different CNVs may be associated with different mechanisms for schizophrenia.

We show that unaffected but high-genetic-risk participants (i.e. carriers of SCZ-associated CNVs) present with smaller cortical surface area, coinciding with previous results shown in individuals with schizophrenia.^10,25^ However, our carriers did show thicker cortex, opposing previous results in clinical samples.^10,25,26^. Interestingly, previous research on 22q11.2 deletions – a CNV highly penetrant for schizophrenia but not observed in our sample – also showed reductions in surface area, but rather widespread thicker cortex in a mixed sample of unaffected and clinically ascertained carriers.^6^ The authors of that research also showed that carriers of the 22q11.2 deletion with psychosis had thinner frontal and temporal cortices but did not differ in surface area from carriers with no psychosis.^6^ Likewise, a recent paper looking into 15q11.2 deletions showed carriers to present smaller surface area but thicker cortices, affecting mainly frontal regions.^27^ Thinning of the cortex in schizophrenia has been associated with illness progression and severity, poor treatment outcome and use of antipsychotic medication,^10,28,29^ whereas surface area has not shown association with illness severity/progression indices,^10^ although admittedly research on surface area is still scarce. Taking these together, cortical thickness would appear to be associated with clinical state, rather than with premorbid risk for schizophrenia, whereas surface area behaves as would be expected from an indicator of risk for schizophrenia, being present – albeit attenuated – in genetically at-risk but unaffected participants. However, this interpretation should be cautiously considered, since owing to their age, our SCZ-associated CNV carriers are very unlikely to develop schizophrenia. Despite both these brain measures being associated with performance IQ (fluid intelligence) and the Townsend deprivation index score in our sample, they did not appear to mediate the association between SCZ-associated CNV carrying status and those phenotypic measures (supplementary Table 6).

From our gyri and individual CNV analyses, it emerges that SCZ-associated CNVs do not uniformly affect the brain, and that not all SCZ-associated CNVs have similar effects on cortical anatomy. Accepting the caveat that individual CNVs have different prevalence rates in our sample and therefore some analyses would be more powered than others, the 15q11.2 deletion appears to affect both surface area and cortical thickness – concurring with previous research^27^ – whereas no other SCZ-associated CNV appears to affect both these brain markers. In fact, the only other two CNVs showing FDR-corrected associations with cortical anatomy are 1q21.1 deletions and 1q21.1 duplications. These reciprocal CNVs have been shown to be associated with head size – microcephaly in deletion carriers and macrocephaly in duplication carriers^30^ – and in fact they demonstrated changes in their association with surface area, but not cortical thickness, when intracranial volume was not included as covariate. Our results suggest that the association between overall head size and these CNVs is mainly driven by their effects on surface area. However, 1q21.1 deletion and duplication, rather than showing a dose effect (affecting the same metric in opposing directions), appear mainly to associate with different metrics: 1q21.1 deletions with thickness and 1q21.1 duplications with surface area. Moreover, the 1q21.1 duplication is the only SCZ-associated CNV in our sample to show positive associations with surface area, all other SCZ-associated CNVs showing only negative associations even with uncorrected *P* < 0.05. The heterogeneity of effects across CNVs was substantiated by the fact that comparing carriers of SCZ-associated CNVs excluding 15q11.2 deletions (the most prevalent CNV in our sample) with non-(CNV) carriers offered substantially different results than the comparison of carriers of 15q11.2 deletions with non-CNV carriers (supplementary Table 7 and Fig. 2), and that the direct comparison between 15q11.2 deletion carriers (*n* = 59) versus other SZ-CNV carriers (*n* = 61) resulted in significant results despite the dramatic reduction in statistical power (supplementary Table 7). We previously showed a rather homogeneous effect across SCZ-associated CNVs on subcortical volumes,^31^ which appears not to be mirrored in the cortex. That result is not surprising, though, in light of previous research showing regional differences in genetic correlation for both cortical thickness^32^ and surface area.^33^ Further replication in larger samples is required, particularly for the less prevalent SCZ-associated CNVs. This will also open the opportunity to investigate how individual SCZ-associated CNVs may associate with potential distinct downstream clinical characteristics, informing a neurobiologically driven patient stratification.

The structural covariance analyses showed higher brain integration with regard to cortical thickness than to surface area. Moreover, the structural covariance topographical distribution across gyri was almost identical in both cortical measures between SCZ-associated CNV carriers and non-carriers, indicating no gross topological differences. However, carriers showed higher thickness covariance compared with non-carriers, this being mainly driven by the 15q11.2 deletion and the 16p13.11 duplication, mostly affecting frontal and parietal cortices. The 1q21.1 duplication also showed some association with increased thickness covariance, although in this case that only survived correction for multiple testing when reducing the number of comparisons via ICA. The pattern of associations, though, was similar to the one shown by the 15q11.2 deletion and the 16p13.11 duplication. Previous reports of increased structural covariance in corticocortical networks in participants with schizophrenia, alongside thinning of the cortex, have been interpreted as an indication of neuronal over-pruning during adolescence.^12^ This over-pruning explanation would not fit with our findings of increased structural covariance in SCZ-associated CNV carriers alongside thickening of the cortex. In keeping with the interpretation of structural covariance as an index of developmental trajectory integration,^9^ our results seem to suggest that changes in thickness occurring in carriers would have more widely affected the brain, whereas changes in surface area might have affected the brain in a more modular manner, causing less interdependency or covariance across gyri. Our study design, however, does not allow us to investigate whether this higher covariance indicates more integrated early thickness development related to cell migration during prenatal development,^29^ deficient neuronal pruning – in this case, lesser pruning – happening uniformly across the brain during the first decades of life^34^ or a general slowing down of the normal tissue loss in latter life if we take into account that our participants were between 45 and 80 years of age.^35^

### Limitations

Some caveats to this study should be noted. Despite including ~17 000 participants, sample size remains a limitation. The advantage of focusing on unaffected participants is obvious as regards avoiding confounders associated with mental disorders (e.g. use of medication). However, this strategy limits sample size because of the high penetrance of SCZ-associated CNVs and therefore statistical power. Future releases of UK Biobank's imaging data – with a final target of 100 000 participants – should allow a more comprehensive investigation of the effects of these SCZ-associated CNVs. Also, we obtained thickness and surface area measures on the basis of the D–K atlas parcellation; however, genetic influences over the brain may not necessarily follow the boundaries established by gyri,^32^ causing a dilution of potential effects. Finally, owing to a recruitment bias in the UK Biobank cohort, this is not a truly representative sample of the general population,^36^ and it includes fewer CNV carriers than would be expected on the basis of previous prevalence data (for example, out of the initial 20 664 sample with T1 data, there were no carriers of the 22q11.2 deletion, and even before applying any of our inclusion criteria the sample included only 7 more carriers of any SCZ-associated CNV). The sample did not include any SCZ-associated CNV carriers positive to schizophrenia, who would have been an interesting comparison group against the unaffected SCZ-associated CNV carriers.

### Implications for future research

Our results endorse that carrying rare risk alleles associated with schizophrenia predicts reductions in cortical surface area in unaffected participants, supporting the association of this metric with genetic liability for the disorder in the absence of potential confounders associated with clinical state. Results for cortical thickness, though, suggest that the thinning of the cortex previously reported in patients is most likely to be a sequela of the disorder, or be unrelated to rare allele risk. Increased covariance for cortical thickness in carriers also indicates larger integration of this measure across gyri, suggesting less modularity in response to potential changes. We also showed an important heterogeneity in the effects of individual SCZ-associated CNVs over cortex, suggestive of distinct neurobiological mechanisms in schizophrenia. Future research should investigate whether risk associated with different SCZ-associated CNVs leads to a shared singular symptom profile or to different symptom profiles under a common clinical diagnostic – but phenotypically heterogeneous – label.

## Data Availability

All data used in this research are publicly available to researchers on application to UK Biobank: www.ukbiobank.ac.uk.

## Supplementary material

For supplementary material accompanying this paper visit https://doi.org/10.1192/bjp.2020.139. click here to view supplementary material
