# Supplementary material for: Effects of genomic copy number variants penetrant for schizophrenia on cortical thickness and surface area in healthy individuals: analysis of the UK Biobank
Source: Br J Psychiatry. 2021 Feb;218(2):104–11. doi: 10.1192/bjp.2020.139 (PMC7844611; doi:10.1192/bjp.2020.139)
Supplement: Supplementary file 1 [file S0007125020001397sup001.zip › S0007125020001397sup002.docx]

Supplemental Table 1. List of schizophrenia associated CNVs with critical region coordinates, calling criteria and number of genes hit. Schizophrenia (SZ) relative risk estimates for each CNV taken from Rees *et al* JAMA Psychiatry;. 2016; 73(9):963-969, and schizophrenia odds ratios taken from Marshall *et al* Nat Genet. 2017;49(10)27-35.

| **CNV (n carriers)** | **Locus** | **Critical/Unique**  **Sequence Region (hg19)** | **N Bialleleic**  **Axiom probes** | **PSD Genes (from 685 PSD genes)** | **Calling criteria** | **N genes** | **SZ relative risk** | **SZ odds ratio** |
| --- | --- | --- | --- | --- | --- | --- | --- | --- |
| 1q21.1del (n=9) | 1q21.1 | chr1:146,527,987-147,394,444 | 289 |  | Size >50% of critical region | 9 | 5.6 | 5.42 |
| 1q21.1dup (n=11) | 1q21.1 | chr1:146,527,987-147,394,444 | 289 |  | Size >50% of critical region | 9 | 2.1 | 6.28 |
| *NRXN1*del (n=6) | 2p16.3 | chr2:50145643-51259674 | 235 | *NRXN1* | Exonic deletions | 1 | 4.2 | 5.87 |
| 3q29del (n=0) | 3q29 | chr3:195,720,167-197,354,826 | 627 | *DLG1* | Size >50% of critical region | 28 | 13.5 | Inf |
| WBSdup (n=1) | 7q11.23 | chr7:72,744,915-74,142,892 | 349 | *STX1A* | Size >50% of critical region | 26 | 4.3 | Inf |
| 15q11.2del BP1-BP2 (n=59) | 15q11.2 | chr15:22,805,313-23,094,530 | 145 | *CYFIP1* | Size >50% of critical region | 5 | 1.7 | 1.8 |
| PWS/ASdup (n=0) | 15q11-q13 | chr15:22,805,313-28390339 | 1807 | *CYFIP1* | Full critical region, ~4Mbp | 116 | 29.2 | Inf |
| 15q13.3del BP4-BP5 (n=0) | 15q13.3 | chr15:31,080,645-32,462,776 | 466 |  | Size >50% of critical region | 8 | 4.2 | 10.55 |
| 16p13.11dup (n=25) | 16p13.11 | chr16:15,511,655-16,293,689 | 349 | *MYH11* | Size >50% of critical region | 7 | 1.7 | 1.49 |
| 16p12.1del (n=8) | 16p12.1 | chr16:21,950,135-22,431,889 | 168 | *UQCRC2* | Size >50% of critical region | 8 | 3.4 | 3.22 |
| 16p11.2dup (n=2) | 16p11.2 | chr16:29,650,840-30,200,773 | 193 | *ALDOA,CORO1A,MAPK3,TAOK2* | Size >50% of critical region | 30 | 8.6 | 13.8 |
| 16p11.2distaldel (n=0) | 16p11.2 | chr16:28,823,196-29,046,783 | 103 | *TUFM* | Size >50% of critical region | 11 | 1.2 | 12.68 |
| 22q11.2del (n=0) | 22q11.2 | chr22:19,037,332-21,466,726 | 843 | *PI4KA,SEPT5* | Size >50% of critical region | 61 | 21.6 | Inf |

Note: total number SZ-CNV carriers (any of the above) equals 120 since two participants carried two of the CNVs included in this table. PSD = Post-synaptic density, WBS = Williams-Beuren syndrome, PWS/AS = Prader-Willi/Angelman syndromes.
