# Supplementary material for: Effects of genomic copy number variants penetrant for schizophrenia on cortical thickness and surface area in healthy individuals: analysis of the UK Biobank
Source: Br J Psychiatry. 2021 Feb;218(2):104–11. doi: 10.1192/bjp.2020.139 (PMC7844611; doi:10.1192/bjp.2020.139)
Supplement: Supplementary file 1 [file S0007125020001397sup001.zip › S0007125020001397sup004.docx]

Supplemental table 5. Association between specific CNV-carrier status with brain surface area and cortical thickness excluding ICV as covariate of interest.

|  | Surface area (mm^2^) | | Cortical thickness (mm) | |
| --- | --- | --- | --- | --- |
| CNV (n carriers) | Total n | Beta* (p value) | Total n | Beta* (p value) |
| SZ-CNV (n=120)  1q21.1 del (n=9)  1q21.1 dup (n=11)  *NRXN1* del (n=6)  15q11.2 del (n=59)  16p13.11 dup (n=25)  16p12.1 del (n=8) | 16,566  16,456  16,459  16,454  16,506  16,473  16,456 | -.020 (.002)  -.037 (5.6e-9)  .012 (.049)  -.002 (.733)  -.009 (.157)  -.004 (.487)  -.014 (.027) | 16,496  16,389  16,391  16,386  16,437  16,404  16,388 | .015 (.035)  .008 (.252)  .001 (.882)  -.001 (.866)  .020 (.005)  -.003 (.629)  .008 (.279) |
