# Supplementary material for: Effects of genomic copy number variants penetrant for schizophrenia on cortical thickness and surface area in healthy individuals: analysis of the UK Biobank
Source: Br J Psychiatry. 2021 Feb;218(2):104–11. doi: 10.1192/bjp.2020.139 (PMC7844611; doi:10.1192/bjp.2020.139)
Supplement: Supplementary file 1 [file S0007125020001397sup001.zip › S0007125020001397sup007.docx]

Supplemental Table 7. Association between fluid intelligence and Townsend deprivation index separately with SZ-CNV carrying status, cortical thickness and surface area, and jointly.

|  | Fluid intelligence | Townsend index |
| --- | --- | --- |
| *Predictors in model** |  |  |
| SZ-CNV carrier status | ß= -0.04, p= 4x10^-7^ | ß= 0.02, p= .019 |
| Cortical thickness | ß= 0.03, p= .002 | ß= -0.02, p= .015 |
| Surface area | ß= 0.12, p= 2x10^-14^ | ß= -0.03, p= .029 |
| SZ-CNV carrier status  Cortical thickness | ß= -0.04, p= 1x10^-5^  ß= 0.03, p= .002 | ß= 0.02, p= .014  ß= -0.02, p= .025 |
| SZ-CNV carrier status  Surface area | ß= -0.04, p= 8x10^-7^  ß= 0.12, p= 2x10^-13^ | ß= 0.02, p= .036  ß= -0.03, p= .024 |

*All models included age, sex, intracranial volume and scan centre as covariates

of no interest
