# Supplementary figures and images for: Effects of genomic copy number variants penetrant for schizophrenia on cortical thickness and surface area in healthy individuals: analysis of the UK Biobank
Source: Br J Psychiatry. 2021 Feb;218(2):104–11. doi: 10.1192/bjp.2020.139 (PMC7844611; doi:10.1192/bjp.2020.139)

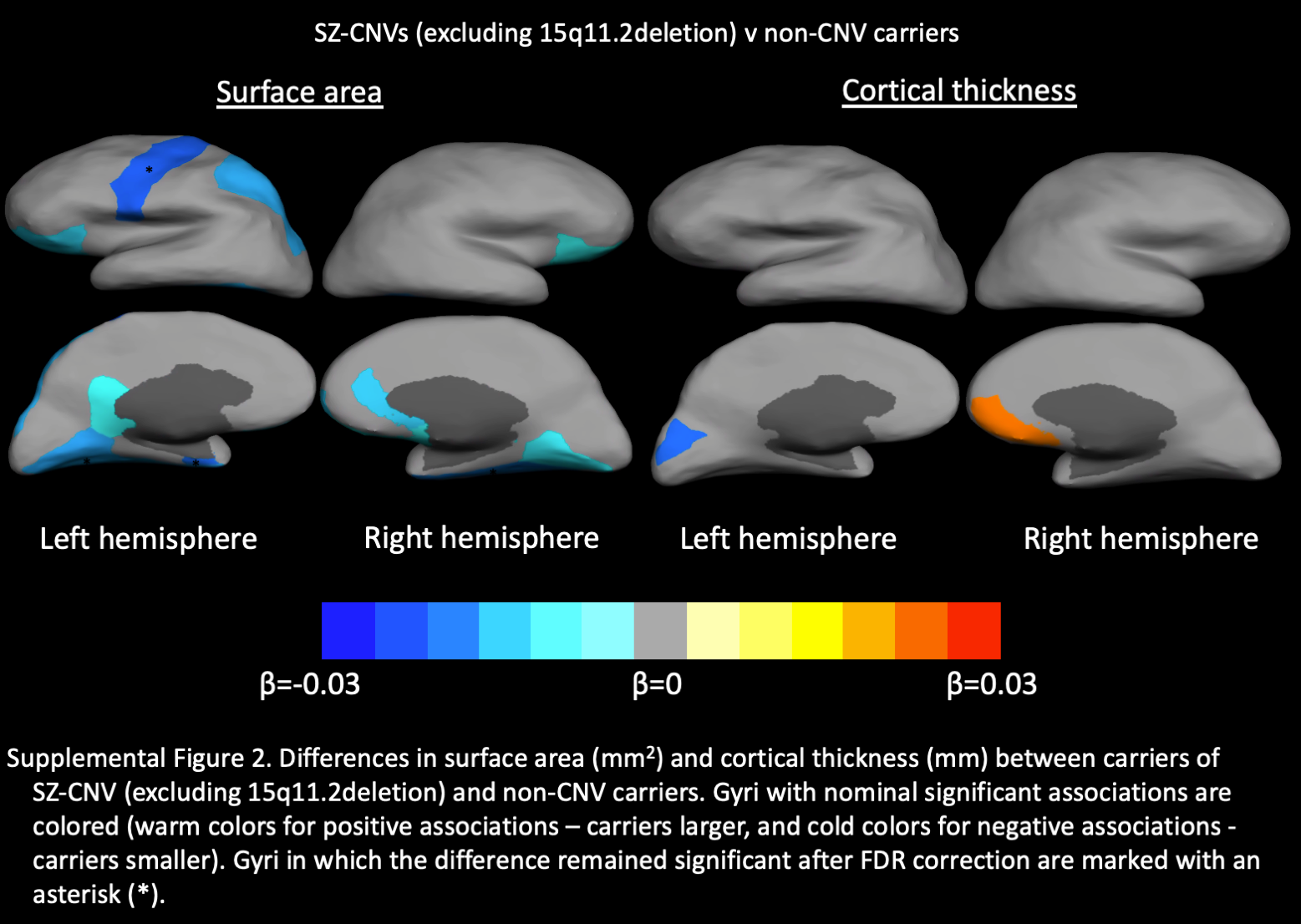

Supplement: Supplementary file 1 [file S0007125020001397sup001.zip › S0007125020001397sup001.tiff]

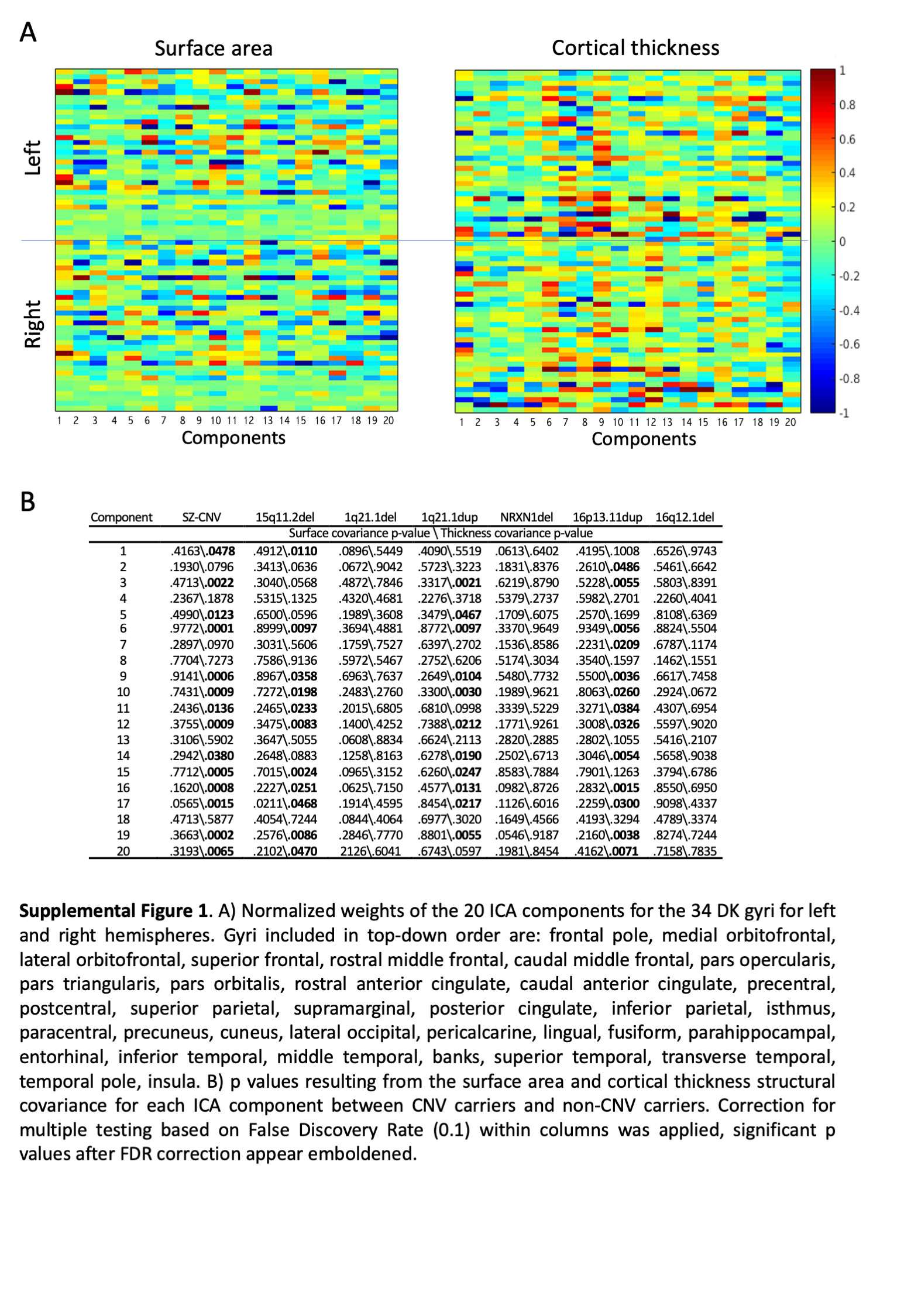

Supplement: Supplementary file 1 [file S0007125020001397sup001.zip › S0007125020001397sup009.tiff]
